# Aerobic exercise decreases interstitial glucose concentrations up to 2 h after exercise in dogs with insulin-treated diabetes mellitus: a preliminary study

**DOI:** 10.3389/fvets.2025.1595952

**Published:** 2025-06-19

**Authors:** Jessica R. Mampe, Darko Stefanovski, Rebecka S. Hess

**Affiliations:** ^1^Department of Clinical Sciences and Advanced Medicine, School of Veterinary Medicine, University of Pennsylvania, Philadelphia, PA, United States; ^2^Department of Clinical Studies-New Bolton Center, School of Veterinary Medicine, University of Pennylvania, Kennett Square, PA, United States

**Keywords:** diabetic, flash glucose monitoring system, FreeStyle Libre, exercise, insulin, hypoglycemia

## Abstract

**Introduction:**

The effect of aerobic exercise on glucose concentration has been reported in healthy normal and over-conditioned dogs and in experimental dog models. However, the effect of aerobic exercise on interstitial glucose concentration (IG) has not been reported in dogs with insulin-treated naturally-occurring diabetes mellitus.

**Objective:**

Determine if aerobic exercise decreases IG in outpatient diabetic dogs.

**Methods:**

Five NPH insulin-treated client-owned diabetic dogs were prospectively enrolled into this interventional longitudinal cohort study. Dogs with a flash glucose monitoring system performed once daily aerobic exercise over 30 min for 7 consecutive days, if IG was ≥ 60 mg/dL during the preceding 12 h of observation. Dogs weighing <10 kg exercised (walked or jogged) for 1.5–2 miles, dogs 10–20 kg exercised for 2–2.5 miles, and dogs >20 kg exercised for 2.5–3 miles. Multivariable mixed-effects linear regression models followed by post-hoc analyses were used to estimate the marginal mean differences between IG 0.5, 1, 1.5, 2, and 4 h after exercise compared with marginal mean baseline IG measured twice over 30 min just before each daily exercise period, which served as the control.

**Results:**

Marginal means (95% confidence intervals) of IG were significantly lower 1.5 h after exercise [188 mg/dL (96-281 mg/dL)] and 2 h after exercise [185 mg/dL (82–287 mg/dL)] compared with marginal mean IG measured just before exercise [223 mg/dL (129–317 mg/dL, *p* = 0.03, *p* = 0.008, respectively)]. Marginal means of IG were not significantly different 4 h after exercise compared with marginal mean IG measured just before exercise.

**Conclusion:**

Our preliminary data suggest that aerobic exercise may reduce IG levels up to two hours following exercise. These findings indicate that exercise could potentially serve as an adjunct approach to managing insulin-treated diabetic dogs in a home setting.

## Introduction

Exercise is an important component of type 1 diabetes mellitus (DM) treatment in humans (1). The benefits of exercise in humans with type 1 DM include a decrease in glycosylated hemoglobin concentration and required insulin dose, improved body condition score, a desired decrease in cholesterol and triglycerides concentration, and improved cardiovascular health (1). However, in humans with type 1 DM exercise increases the risk of severe hypoglycemia with coma (2). Although aerobic exercise is characterized by a decrease in insulin secretion and an increase in glucagon secretion these changes usually do not compensate for increased glucose uptake by muscle, and blood glucose concentration does decrease in most type 1 DM individuals who perform aerobic exercise (1, 3). Unlike aerobic exercise, anerobic exercise usually results in hyperglycemia because above a certain threshold of lactate, glucose output from the liver is greater than glucose uptake by muscles (3). Therefore, in people with type 1 DM, guidelines regarding the timing and content of exercise related carbohydrate intake are tailored to the type of exercise performed (3). Guidelines for the use of continuous glucose monitors before, during, and after exercise in people with type 1 DM are also adjusted to the type, intensity, duration, and timing of exercise, the dose of insulin, and risk of hypoglycemia (4). In humans with type 1 DM it is recommended that during exercise, sensor interstitial glucose concentrations (IG) range between 126 mg/dL and 180 mg/dL, and higher IG are recommended for those with an increased risk of hypoglycemia (4).

A study of healthy normal and over-conditioned dogs that were not treated with insulin reported that 15 min of aerobic exercise performed 30 min after a maintenance or high carbohydrate meal resulted in no significant differences in glucose concentrations (5). The effect of aerobic exercise on blood glucose concentration in dogs with experimentally induced insulin-treated diabetes has also been studied (6). In dogs running on a 15% slope treadmill at a speed of 100 meters/min for 3 h 48–60 h after their last insulin dose, blood glucose concentration decreased significantly for 15–90 min after exercise, and this was associated with increased glucose hepatic output that could not match the increase in glucose clearance (6). In another study, a high concentration somatostatin infusion caused undetectable insulin concentrations in dogs that were exercised for 90 min on a treadmill (7). In this model glucose concentration was unchanged by exercise, and this was explained by a relative decrease in muscle uptake of glucose compared with dogs given insulin, and an increase in hepatic glucose production (7).

The effect of aerobic exercise on glucose concentrations in dogs with naturally-occurring insulin-treated DM has not been reported. The goal of this study was therefore to determine if aerobic exercise decreases IG in outpatient diabetic dogs treated with NPH insulin. It was hypothesized that aerobic exercise decreases IG in NPH-insulin-treated diabetic dogs when exercise is performed during the time period of 8–12 h after insulin administration, at which time IG should be higher than all other time points (8). It was further hypothesized that exercise performed during the time period of 8–12 h after insulin administration would decrease IG to the extent that it is lower after the exercise period, compared with IG measured twice over 30 min just before exercise.

## Materials and methods

Client-owned dogs with naturally-occurring DM that were examined at a veterinary university teaching hospital were prospectively enrolled in this interventional longitudinal cohort study. Inclusion criteria were a diagnosis of DM for at least 4 weeks prior to enrollment, an unchanged insulin type and dose for at least 2 weeks prior to enrollment and eating the same two meals a day at fixed times with no other snacks or treats. Only dogs receiving twice daily subcutaneous Neutral Protamine Hagedorn (NPH) insulin immediately after a meal were enrolled. Exclusion criteria were an episode of diabetic ketoacidosis within 4 weeks of enrollment into the study, treatment with topical or systemic glucocorticoids, cyclosporine, tramadol, trilostane, mitotane, thyroid supplementation, antibiotics, or anticonvulsants, and any clinical signs indicative of illness including vomiting, diarrhea, regurgitation, coughing, and increased respiratory rate or effort. Dogs treated with furosemide, spironolactone, enalapril, benazepril, lisinopril, pimobendan, torsemide, sildenafil, or sacubitril/valsartan were excluded to avoid inclusion of dogs with cardiac disease. Additionally, dogs with recent exposure to acetaminophen, dopamine, icodextrin, salicylates, and ascorbic acid or dogs requiring anesthesia, radiography, computed tomography, or magnetic resonance imaging during the 8-day study period were excluded because the flash glucose monitoring system (FGMS) sensor can be affected by these medications or procedures. Dogs with clinical signs suggestive of hypoglycemia such as weakness, incoordination, disorientation, collapse, or seizures were also excluded. Finally, dogs in which IG was < 60 mg/dL during a pre-screening period which consisted of the 12 h immediately preceding the first day in which exercise was introduced, were excluded from the study to avoid inclusion of dogs who could develop exercise induced hypoglycemia. Dogs were kept on the same insulin type and dose and the same amount and type of diet for the entire 8-day study period. The University Privately Owned Animal Protocol Committee and the Institutional Animal Care and Use Committee approved the study and informed consent form, and owners signed the written informed consent form prior to enrollment.

On the day of enrollment, a 14-day FGMS sensor (FreeStyle Libre 14 day, Abbott) was placed on the dorsal-lateral shoulder of the dog in a standardized and previously reported fashion (9). Concentrations of IG were recorded every 15 min throughout the study period, barring periods of sensor malfunction or unintentional temporary removal. The sensor was left in place for the 8-day study period. If the sensor fell off or was removed by the dog before the end of the 8-day study period, it was replaced with another sensor. At the end of the study, owners were given the option of removing the FGMS or keeping it in place for a total of 14 days after it was placed. Biochemical hypoglycemia was defined as an IG < 60 mg/dL. Data recorded by the FGMS during the first 12 h after sensor placement were reviewed to determine if any IG measurements were <60 mg/dL. If no IG < 60 mg/dL were reported during the 12-h pre-screening period, owners were instructed to start once-daily aerobic exercise for 7 consecutive days. To decrease the risk of inducing hypoglycemia, aerobic exercise was performed during the time period of 8–12 h after insulin administration because the mean time to reach minimum blood glucose concentration in NPH treated diabetic dogs is 4.9 ± 3.0 h after insulin administration (8). Exercise was timed such that the exercise period ended within 12 h from the last insulin treatment and feeding, and before the scheduled time of the next insulin treatment and feeding. Dogs were exercised for 30 min daily for 7 days during the time period of 8–12 h after morning feeding and insulin administration, and their IG during and after aerobic exercise was compared with baseline IG measured twice over 30 min just before each daily exercise. Baseline IG measured twice over 30 min just before each daily exercise period served as controls. Two baseline IG readings were measured prior to exercise to enhance the reliability and accuracy of these values as controls.

Dogs weighing <10 kg exercised (walked or jogged) for 1.5–2 miles (2.4–3.2 kilometers) per aerobic exercise period, dogs 10–20 kg exercised for 2–2.5 miles (3.2–4.0 kilometers) per aerobic exercise period, and dogs >20 kg exercised for 2.5–3 miles (4.0–4.8 kilometers) per aerobic exercise period. Each dog was allowed up to a 0.5-mile (0.8 kilometer) variation per exercise period, as long as the aerobic exercise was performed within the allotted 30-min exercise period. The start and end-time of each 30-min aerobic exercise period as well as the distance traveled by the dog during the aerobic exercise period were recorded with the use of the smart phone application Amiko - Dog Walk Tracker (10). This easy to use application utilizes a global positioning system to track the route and distance traveled, and it only requires the use of 1 start and stop button. At the end of the study period, owners were asked to share screen shots of each daily exercise period, and these screen shots included the precise timing and length of each of the 7 daily aerobic exercise periods. Owners also recorded the time of meals and insulin administration, twice daily, throughout the study. Owners were asked to fill out a standardized client-questionnaire regarding their dog’s clinical signs including polyuria, polydipsia, appetite, and any clinical signs suggestive of hypoglycemia such as weakness, incoordination, disorientation, collapse, or seizures on the day of enrollment and on the day of study completion. The questionnaire also included questions regarding medications, diet, insulin type and dose, times of meals and insulin administration, to verify that no changes other than daily aerobic exercise were introduced during the study period.

### Statistical analysis

Descriptive analyses included computation of medians, minimum, and maximum for continuous variables, which were not normally distributed, as determined visually and by the skewness and kurtosis tests. Frequency counts and percentages were used to report categorical variables.

For the purpose of inference statistical analysis, marginal mean IG as the primary outcome were assessed using a mixed-effect linear regression model with the period after exercise in hours (0.5 h after exercise, 1 h after exercise, 1.5 h after exercise, 2 h after exercise, and 4 h after exercise) as the major fixed effect. Observed IG values were grouped in 6 sequential intervals (including baseline) named after the last value in the interval and with times signifying time after exercise in hours. The only exception is the baseline which was obtained before exercise. For example, the first interval starts right after the exercise and ends at 30 min after exercise and was named 0.5 h interval. The next interval starts right after the first one and ends with 1 h after exercise. Please note that within this interval there were multiple values obtained. Since the intervals were considered a categorical variable, the naming of the intervals does not have an impact on the analysis. Since we are using a repeated measures mixed-effect model, the values were not average within the interval but rather as just labeled to belong in the interval. Random effects were set on the level of individual animal. Robust variance estimation (sandwich estimator) was used to adjust for heteroskedasticity. Post-hoc, marginal (model-adjusted) means and effects were estimated. When using statistical models with 2 or more independent variables (fixed or random-effects) to explain a given outcome (the dependent variable or IG in this case), the marginal mean for a given outcome in respect to 1 of the independent variables is the mean of the dependent variable averaged across all levels of the other independent variables (11). The least significant difference method was used to correct for multiple pairwise comparisons. There are two main reasons to use this method. First, it is the default STATA correction method for post-hoc comparisons. Second, it is very sensitive, and it detects differences that otherwise may be missed with more conservative methods, like the Bonferroni correction, especially when dealing with very few pairwise comparisons, such as in this study (12). Marginal means and effects are reported with 95% confidence intervals. All analyses were conducted with Stata 18MP, StataCorp, College Station, TX, with two-sided tests of hypotheses and a *p*-value < 0.05 as the criterion for statistical significance.

## Results

Five of 185 diabetic dogs (3%) examined during the study period satisfied the inclusion and exclusion criteria, and all were enrolled in the study. All of the dogs enrolled in the pre-screening period went on to complete the study because none of the dogs had an IG < 60 mg/dL during the 12-h pre-screening period. Reasons for exclusion of 181 of 185 (97%) diabetic dogs examined during the study period included concurrent illness documented in 166 of 181 dogs (92%), administration of medication that warranted exclusion in 106 of 181 dogs (59%), use of an insulin other than NPH in 55 of 181 dogs (30%), owners who did not reply to attempted contact by the research team or declined to participate in the study in 23 of 181 dogs (13%), death or euthanasia by the time the owners were contacted by the research team in 5 of 181 (3%) dogs, and a recent hospitalization for hypoglycemia in 1 dog (0.5%). Some dogs had more than one reason for exclusion. The median age of study dogs was 10 years (range, 8–17 years), median weight was 7.6 kg (range, 5.5–42 kg), and median body condition score was 6 (range, 4–8) at the time of enrollment (13). The median NPH insulin dose which was unchanged throughout the study period was 0.64 units/kg (range, 0.36–0.89 units/kg) given subcutaneously twice daily. Breeds included Chihuahua (2/5, 40%), mixed breed (2/5, 40%), and Labrador Retriever (1/5, 20%). Sex included neutered females (3/5, 60%) and neutered males (2/5, 40%). Diets included Royal Canin Gastrointestinal Low Fat®, Hill’s Healthy Advantage Adult Canine®, Purina Canine DM®, Royal Canin Canine Glycobalance®, and Purina ProPlan Chicken and Rice® in one dog each.

The FGMS sensors dislodged or malfunctioned 3 times throughout the study period. In one dog, the sensor fell off twice. It first fell off 15 h after the initial placement before any exercise was performed and was replaced 10 h later. The second sensor on the same dog stopped working 4.5 h after the end of the exercise period on day 2 and was replaced the following morning before the next scheduled exercise period. In another dog, the sensor stopped working 3.5 h after the end of the exercise period on day 5 and was replaced the following day. Each exercise session lasted the intended 30 min, except for 1 session that ended prematurely at 25 min due to adverse weather conditions. The median time at which aerobic exercise began was 10.5 h (range, 8.8–11.4 h) after the morning insulin dose and feeding. The median time between the end of aerobic exercise and feeding dinner was 42.5 min (range, 1–157 min). There were no adverse events reported during the study period, and none of the dogs exhibited clinical signs suggestive of hypoglycemia or illness.

Marginal means and 95% confidence intervals of IG concentrations measured 1.5 h after exercise [188 mg/dL (96-281 mg/dL)] and 2 h after exercise [185 mg/dL (82-287 mg/dL)] were significantly lower than marginal mean baseline IG measured twice over 30 min just before each daily exercise [223 mg/dL (129-317 mg/dL, *p* = 0.03, *p* = 0.008, respectively)]. Marginal means of IG were not significantly different 4 h after exercise compared with marginal mean IG measured just before exercise. These results are also reported in Table 1.

**Table 1:** Marginal means and 95% confidence intervals of interstitial glucose concentrations at different sequential time intervals after aerobic exercise compared with marginal mean interstitial glucose concentrations measured twice over 30 min just before each daily exercise (223 mg/dL, 95% confidence interval 129–317 mg/dL) in neutral protamine Hagedorn insulin-treated diabetic dogs.

Period of interstitial glucose concentrations measurement	Marginal mean (mg/dl)	95% confidence interval (mg/dl)	*p* value
0.5 h after exercise	223*^,§^	134–313	0.9
1 h after exercise	205^	111–299	0.2
1.5 h after exercise	188*^,^^	96–281	0.03
2 h after exercise	185^§^	82–287	0.008
4 h after exercise	201	85–316	0.2

*Marginal mean interstitial glucose concentrations significantly different (*p* = 0.001).

^§^Marginal mean interstitial glucose concentrations significantly different (*p* < 0.0001).

^^^Marginal mean interstitial glucose concentrations significantly different (*p* = 0.004).

## Discussion

Marginal mean IG concentrations measured 1.5 h after aerobic exercise and 2 h after aerobic exercise were significantly lower than marginal mean baseline IG measured twice over 30 min just before each daily exercise. However, marginal mean IG measured 4 h after aerobic exercise, was no longer significantly different than marginal mean IG measured at baseline. Therefore, the IG lowering effect of 0.5 h of aerobic exercise in NPH insulin-treated diabetic dogs lasts for up to 2 h after exercise and wears off by 4 h after exercise. Dogs were exercised in the evening, and nighttime IG are significantly higher than daytime IG in insulin-treated dogs with DM (9). Therefore, the nighttime increase in IG could have masked a component of an exercise induced decrease in IG. This study design consideration was implemented to decrease the risk of hypoglycemia. However, a nighttime increase in IG could have masked a longer duration and larger magnitude of an aerobic exercise induced decrease in IG, than the duration and magnitude of IG decrease reported here.

Hypoglycemia can be fatal in dogs, and every attempt was made to avoid the risk of hypoglycemia (14). It was therefore recommended that dogs exercise during the time period of 8–12 h after insulin administration and after the time of peak NPH action, to decrease the risk of hypoglycemia (8). Additionally, a mild to moderate aerobic exercise plan was introduced to avoid the risk of exercise induced hypoglycemia. Indeed, none of the study dogs had clinical signs suggestive of hypoglycemia. However, veterinarians and owners must be mindful of the risk of exercise induced hypoglycemia, monitor dogs carefully during and up to 2 h after exercise, and consider providing a snack at the time of exercise or during the 2 h after exercise, to prevent the risk of hypoglycemia. This study facilitates follow-up studies that could explore specific guidelines for aerobic exercise in dogs with insulin-treated DM. Such studies could investigate the effect of longer or more intense exercise regimens on IG glucose, as well as the effect of exercise timing and types of snacks and insulin products in dogs engaging in aerobic exercise. Such studies could help veterinarians, pet owners, and dog trainers make sound recommendations on how to safely add aerobic exercise as an adjunct treatment to insulin in outpatient dogs with DM.

Inherent to the use of the FGMS in dogs is the need to account for inaccuracies (9, 15, 16). Inaccuracies of the FGMS and other continuous glucose monitoring devices are exacerbated in humans with type 1 DM during aerobic exercise (17). In a study of 23 people with insulin-treated type 1 DM, IG measured by the FGMS during 6 days of intense mountain biking including altitude changes and 6 days of normal daily activities, were compared to capillary glucose concentrations (17). Only 59% of IG readings were within 20% of capillary glucose concentrations during exercise compared with 83% during normal daily activities (17). The study concluded that users and care givers should be aware of the risk of decreased FGMS accuracy during intensive exercise (17). Consensus and position statements authored and endorsed by the European Association for the Study of Diabetes, the International Society for Pediatric and Adolescent Diabetes, the Juvenile Diabetes Research Foundation, and the American Diabetes Association regarding exercise in humans with type 1 DM do recommend the use of continuous glucose monitors, including the FGMS, while recognizing their inaccuracies (1, 4). Future validation studies of FGMS readings obtained during aerobic exercise in dogs with insulin-treated DM could refine our understanding and ability to interpret these inaccuracies in dogs.

This study included IG measurements only, and no validation blood glucose concentration measurements because the 14-day FGMS sensor has been extensively validated in dogs (15, 18). It is not unusual for studies of diabetic dogs to report IG only (9, 19). Given the wide use and acceptance of the FGMS as a monitoring tool in dogs with spontaneous diabetes mellitus, the Institutional Animal Care and Use Committee might not grant approval for a study subjecting dogs to venipuncture for the purpose of validating an already validated methodology. However, specific validation studies in dogs performing aerobic exercise could be needed to strengthen our findings.

Owner compliance and accurate reporting of feeding times, insulin administration times, length, duration, intensity, and timing of exercise, are important for accurate and meaningful interpretation of IG changes associated with exercise. In our study, owner compliance was excellent, possibly due to the ease of use of the Amiko - Dog walk tracker (10). In addition to easy and accurate reporting of the exercise period characteristics, owner compliance was excellent in that all exercise sessions lasted the required 30 min, except for one session that lasted only 25 min due to adverse weather conditions. Owner compliance also was excellent in regard to record keeping of the time of feeding and insulin administration. This excellent owner compliance bodes well for future studies of the effect of different aerobic exercise regimens and potential introduction of snacks in outpatient dogs with insulin-treated DM.

Our study has several limitations. The main limitation is the small sample size of only 5 dogs. The enrollment rate was low due to the strict inclusion criteria. The exercise lasted only 7 days, so only a short-term effect of exercise on IG levels was evaluated. The exercise regimen of each dog was uniform throughout the study, and although a 0.5-mile (0.8 km) variation per exercise period was allowed, different intensities, durations, or types of exercise were not examined. Additionally, respiratory rate and heart rate were not measured during exercise, and the intensity of exercise in each dog is not known. Another important limitation is that a dog’s body condition score or baseline fitness level was not used as an inclusion or exclusion criterion in this study. The variable BCS and baseline fitness levels may have impacted the effect of exercise on IG levels differently for each dog. The authors feel that any attempt to recruit dogs of similar size, body condition score, or fitness level would have further inhibited successful enrollment of dogs into this study. If each diabetic dog in the study was otherwise healthy with no comorbidities or medications listed in the exclusion criteria, and they were orthopedically sound enough to participate in 30 min of daily exercise for 1 week, they were enrolled regardless of body condition score and fitness. Enrolling dogs with varying body condition scores may more accurately represent the canine diabetic population as a whole. The dogs included in the study had no concurrent illnesses, but many dogs with DM do have concurrent illness, that could require different considerations and a different study design (20). Dogs with concurrent illness were excluded to decrease the risk of exercise induced complications such as heart failure or dehydration. Additionally, in order to increase the homogeneity of the study population and increase the likelihood that changes in IG were observed because of the exercise intervention and not because of fluctuations in other variables within the population, only dogs receiving NPH insulin were included. Therefore, the findings of this study might relate only to NPH insulin-treated diabetic dogs with no concurrent illness and might not be generalizable to the diabetic dog population at large. Diet was not standardized, and snacks were not provided, and both could be required in future studies to refine the understanding of aerobic exercise induced decrease in IG. Due to the small sample size, strict inclusion criteria, and use of NPH insulin only, our results may not be generalizable to the overall diabetic dog population. Our results and conclusions should only be considered preliminary, and applicable to the study population reported. Future studies of diabetic dogs receiving other types of insulin (porcine lente, glargine, degludec, etc.) and food, as well as studies that include diabetic dogs with concurrent illness are needed to determine the effect of exercise on IG in these other populations of diabetic dogs. Future studies that more specifically define and measure exercise intensity, and those that evaluate exercise at varying intensities and durations, may further clarify the understanding of the effects of exercise on IG levels in diabetic dogs. Longer study durations are also needed to evaluate the long-term effect of exercise on IG levels in diabetic dogs, and to further evaluate how IG levels may change as an individual dog’s BCS or fitness improves. Future studies may also require the use of newer FGMS, and results could be different than the ones obtained in our study, which used the 14-day FGMS sensor.

In conclusion, aerobic exercise performed during the time period of 8–12 h after feeding and NPH insulin administration significantly decreased marginal mean IG 1.5 and 2 h after exercise. The exercise induced change in IG was successfully detected with the 14-day FGMS which can be used in future studies to improve the understanding of the effects of aerobic exercise on IG in outpatient dogs with insulin-treated DM. This preliminary data maybe potentially used by owners and clinicians caring for dogs with insulin-treated DM to optimize exercise as an adjunct mode of treatment.

## Data Availability

The raw data supporting the conclusions of this article will be made available by the authors, without undue reservation.
